# Non-knee-spanning muscles contribute to tibiofemoral shear as well as valgus and rotational joint reaction moments during unanticipated sidestep cutting

**DOI:** 10.1038/s41598-017-19098-9

**Published:** 2018-02-06

**Authors:** Nirav Maniar, Anthony G. Schache, Prasanna Sritharan, David A. Opar

**Affiliations:** 1 School of Exercise Sciences, Australian Catholic University, Melbourne, Australia; 2 Department of Mechanical Engineering, The University of Melbourne, Melbourne, Australia; 3 Sports and Exercise Medicine Research Centre, La Trobe University, Melbourne, Australia

## Abstract

Anterior cruciate ligament (ACL) injuries are a burdensome condition due to potential surgical requirements and increased risk of long term debilitation. Previous studies indicate that muscle forces play an important role in the development of ligamentous loading, yet these studies have typically used cadaveric models considering only the knee-spanning quadriceps, hamstrings and gastrocnemius muscle groups. Using a musculoskeletal modelling approach, we investigated how lower-limb muscles produce and oppose key tibiofemoral reaction forces and moments during the weight acceptance phase of unanticipated sidestep cutting. Muscles capable of opposing (or controlling the magnitude of) the anterior shear force and the external valgus moment at the knee are thought to be have the greatest potential for protecting the anterior cruciate ligament from injury. We found the best muscles for generating posterior shear to be the soleus, biceps femoris long head and medial hamstrings, providing up to 173N, 111N and 77N of force directly opposing the anterior shear force. The valgus moment was primarily opposed by the gluteus medius, gluteus maximus and piriformis, with these muscles providing contributions of up to 32 Nm, 19 Nm and 21 Nm towards a knee varus moment, respectively. Our findings highlight key muscle targets for ACL preventative and rehabilitative interventions.

## Introduction

Anterior cruciate ligament (ACL) injury is a burdensome condition due to potential surgical requirements, substantial convalescence and rehabilitation time^1^, and associated financial costs to individuals and the healthcare system^2^. ACL injury has also been shown to be associated with an increased risk of early onset knee osteoarthritis, especially if accompanied by meniscal injury^3^. Consequently, knowledge regarding the mechanical factors related to ACL injury and injury risk are needed to develop effective prophylactic strategies.

Whilst the primary role of the ACL is to resist anterior translation of the tibia relative to the femur^4^, both cadaveric and modelling studies have shown that frontal and transverse plane knee mechanics can also influence ACL loading^5–8^. In the frontal plane, a greater ‘external’ knee valgus or varus moment has the potential to increase load on the ACL^5,8^. However, knee valgus has been reported to be the more common mechanism of injury in video analysis studies^9–11^. In the transverse plane, an ‘external’ moment causing internal rotation of the tibia with respect to the femur has been found to expose the ACL to higher loads than an ‘external’ moment causing external rotation of the tibia with respect to the femur^5,8^. Moreover, non-sagittal plane knee joint moments have been shown to have the greatest influence on ACL loading when they occur simultaneously and especially in conjunction with an anterior shear force^5,7,8,12^. A better understanding regarding the development of these critical knee joint loads could therefore be beneficial for improving ACL preventative and rehabilitative strategies.

Muscles produce forces that can cause and oppose these critical knee joint loads. For example, the quadriceps generates an anterior tibiofemoral shear force which is directly opposed by the ACL^13^. In contrast, the hamstrings have the potential to mitigate anterior tibiofemoral shear forces thereby working with the ACL to control the amount of anterior translation at the knee joint^13,14^. Despite the amount of research completed to date, existing knowledge regarding biomechanical variables associated with high loading of the ACL is still quite limited. No studies have investigated which muscles contribute most substantially towards critical knee joint loads during high injury risk tasks such as unanticipated cutting. Furthermore, through “dynamic coupling”, any muscle in the body can potentially induce an acceleration of any segment in the body^15^. For example, it is possible that certain hip muscles can influence knee joint loads during rapid change in direction tasks. Ignoring the role of the hip muscles may mean that some valuable information that could be used to guide preventative and rehabilitative interventions has been overlooked.

Musculoskeletal modelling enables the cause-effect relationships between muscle forces and joint loads during high injury risk tasks to be evaluated^16^. Subsequently, the purpose of this study was to investigate the role of the major lower-limb muscles on key tibiofemoral loading parameters associated with ACL injury during an unanticipated sidestep cut. Specifically, we used a computational musculoskeletal modelling approach to predict lower-limb muscle contributions to the knee joint anteroposterior shear force as well as the frontal and transverse plane moments. Our primary interest was to identify which muscles have the greatest capacity to control/minimise the anterior shear force as well as the knee valgus and internal rotation moments, as the function of such muscles could then be targeted in ACL prevention programs.

## Methods

### Participants

Eight recreationally active healthy males (age: 27 ± 3.8 years; height: 1.77 ± 0.09 m; mass: 77.6 ± 12.8 kg) volunteered to participate in this study. All participants had no current or previous musculoskeletal injury likely to influence their ability to perform the required tasks. All participants provided written informed consent to participate in the study. Ethical approval was granted by the Australian Catholic University Human Research Ethics Committee (approval number: 2015-11 H), and the study was carried out in accordance with the approved guidelines.

### Instrumentation

Three-dimensional marker trajectories were collected at 200 Hz using a nine camera motion analysis system (VICON, Oxford Metrics Ltd., Oxford, United Kingdom). Ground reaction forces were collected via two AMTI OR6-6-2000 ground-embedded force plates (Advanced Mechanical Technology Inc., Watertown, MA, USA) sampling at 1000 Hz. Surface electromyographic (EMG) data were collected at 1000 Hz from 10 lower-limb muscles on the dominant leg (defined as the kicking leg; right side for all participants) via two wireless EMG systems (Noraxon, Arizona, USA; Myon, Schwarzenberg, Switzerland).

### Procedures

All participants were barefoot during the completion of all tasks, which allowed exposure of the foot for marker placement and kept the foot-ground interaction consistent across all participants. The skin was prepared for surface EMG collection by shaving, abrasion and sterilisation. Circular bipolar pre-gelled Ag/AgCl electrodes (inter-electrode distance of 2 cm) were then placed on the vastus lateralis and medialis, rectus femoris, biceps femoris, medial hamstrings, medial and lateral gastrocnemius, soleus, tibialis anterior and peroneus longus muscles in accordance with Surface Electromyography for the Non-Invasive Assessment of Muscle (SENIAM) guidelines^17^. EMG-time traces during forceful isometric contractions were visually inspected to verify the correct placement of the electrodes and to inspect for cross-talk. Forty-three 14 mm retroreflective markers were affixed to various anatomical locations on the torso (sternum, the spinous process of the 7^th^ cervical vertebra, the spinous process of a mid-thoracic vertebra, the tip of each acromion), pelvis (anterior and posterior superior iliac spines), upper-limbs (medial and lateral elbow and distal radius and ulna) and lower-limbs (medial and lateral femoral epicondyles, medial and lateral malleoli, first and fifth metatarsophalangeal joints, calcaneus and three additional markers on each shank and thigh) of each participant.

Each participant completed two unanticipated change of direction tasks on their dominant leg. Participants were required to perform two single leg hops for a standardised distance of 1.35 m, and then as quickly as possible cut to the left (45° sidestep cut) or to the right (45° crossover cut) upon landing from the second hop. We used a hopping approach based on prior research^18^ because it allows speed and foot placement on the force plate to be well controlled across participants relative to a running approach. The direction of travel was randomly dictated by a set of timing gates that delivered a light signal ~450 ms prior to initial contact on the force plates. Floor markings were used to indicate the starting point, the hop landing targets and the required 45° angle from the force plates for the cutting direction. A successful trial required that the participant completed the task correctly with the entire foot landing within the force plate. This protocol produced approach velocities (2.24 ± 0.15 m/s) and cutting angles (41 ± 2°) that were consistent with characteristics reported during ACL injuries^19^. Note that we only analysed sidestep cuts in this investigation, as this task has been most commonly associated with injury to the ACL^9,10,19,20^.

### Data processing

Marker trajectories were low-pass filtered using a zero-lag, 4^th^ order Butterworth filter with a cut-off frequency of 8 Hz. This cut-off frequency was determined via a residual analysis. Ground reaction forces were filtered using the same filter and cut-off frequency as the marker data based on published recommendations^21^. EMG data were corrected for offset, high-pass filtered (20 Hz), full-wave rectified and low-pass filtered (6 Hz) using a zero-lag, 4^th^ order Butterworth filter to obtain a linear envelope. EMG data were normalised to the peak amplitude obtained in each trial.

### Musculoskeletal modelling

A 37 degree-of-freedom (DOF) full-body musculoskeletal model, with 80 musculotendon actuators (lower body) and 17 torque actuators (upper body)^22^, was used to perform the musculoskeletal simulations in OpenSim^16^. Each hip was modelled as a 3-DOF ball and socket. Each knee was modelled as a 1-DOF hinge, with other rotational (valgus/varus and internal/external rotation) and translational (anteroposterior and superior-inferior) movements constrained to change as a function of the knee flexion angle^23^. A pin joint was used to represent the ankle (talocrural) joint. The head-trunk segment was modelled as a single rigid segment, articulating with the pelvis via a 3-DOF ball and socket joint. Each upper limb was characterised by a 3-DOF ball and socket shoulder joint and single-DOF elbow and radioulnar joints. The subtalar, metatarsophalangeal, and wrist joints were locked^22^. The generic model was scaled to each participant’s individual anthropometry as determined during a static trial. An inverse kinematics algorithm was used to calculate joint angles by means of a weighted least-squares optimisation that minimised the difference between model and experimental marker positions^24^. Inverse dynamics was used to obtain the joint moments acting about each modelled DOF. Muscle forces were obtained via a static optimisation algorithm, which decomposed the joint moments into individual muscle forces by minimising the sum of muscle activations squared, taking into account the physiological force-length-velocity properties^25^ of the musculotendinous units. This method of muscle force estimation is computationally efficient and has been used to predict muscle forces in similar high impact movements^26–28^. Note that the maximum isometric force of each actuator was increased 3-fold from the standard model, similar to another study that investigated high impact manoeuvres^26^.

The measured ground reaction forces were decomposed into individual muscular contributions by means of a pseudo-inverse-based approach^28–30^. Each muscle’s contribution to the joint reaction forces and moments about the knee were then computed by applying each muscle’s force and contribution to the ground reaction force in isolation and resolving the dynamical equations of motion. The knee joint reaction forces and moments represent the forces and moments that the knee joint experiences as a consequence of all motions and forces in the model, including muscles and other actuators. These parameters differ somewhat from the inverse dynamics based outputs used with the static optimisation algorithm to calculate muscle forces.

### Outcome variables

Outcome variables of interest were each muscle’s contribution to the tibiofemoral anteroposterior shear joint reaction force as well as the frontal and transverse plane joint reaction moments, as these variables have been shown to be associated with higher ACL loads and/or injury^5,6^. Since ACL injuries occur promptly after initial contact^9^, we limited our analysis to the weight acceptance phase (period of stance from foot-strike to the first trough in the raw vertical ground reaction force) as per previous research^31,32^. Muscular contributions were grouped according to function consistent with a prior approach^33^, except where these muscles had opposing effects on the key tibiofemoral loading parameters. For example, the biceps femoris long head and medial hamstrings (i.e. semimembranosus and semitendinosus) have opposing transverse plane actions at the knee, hence the biarticular hamstrings were not grouped together (see Supplementary Table S1 for all functional groupings). Note that we only report on major muscle groups, and did not report on any muscle that was not found to make a meaningful contribution to any of the three key knee reaction forces or moments (see Rajagopal *et al*.^22^ for all musculotendinous actuators included in the model).

### Validation and verification

**Figure 1:**
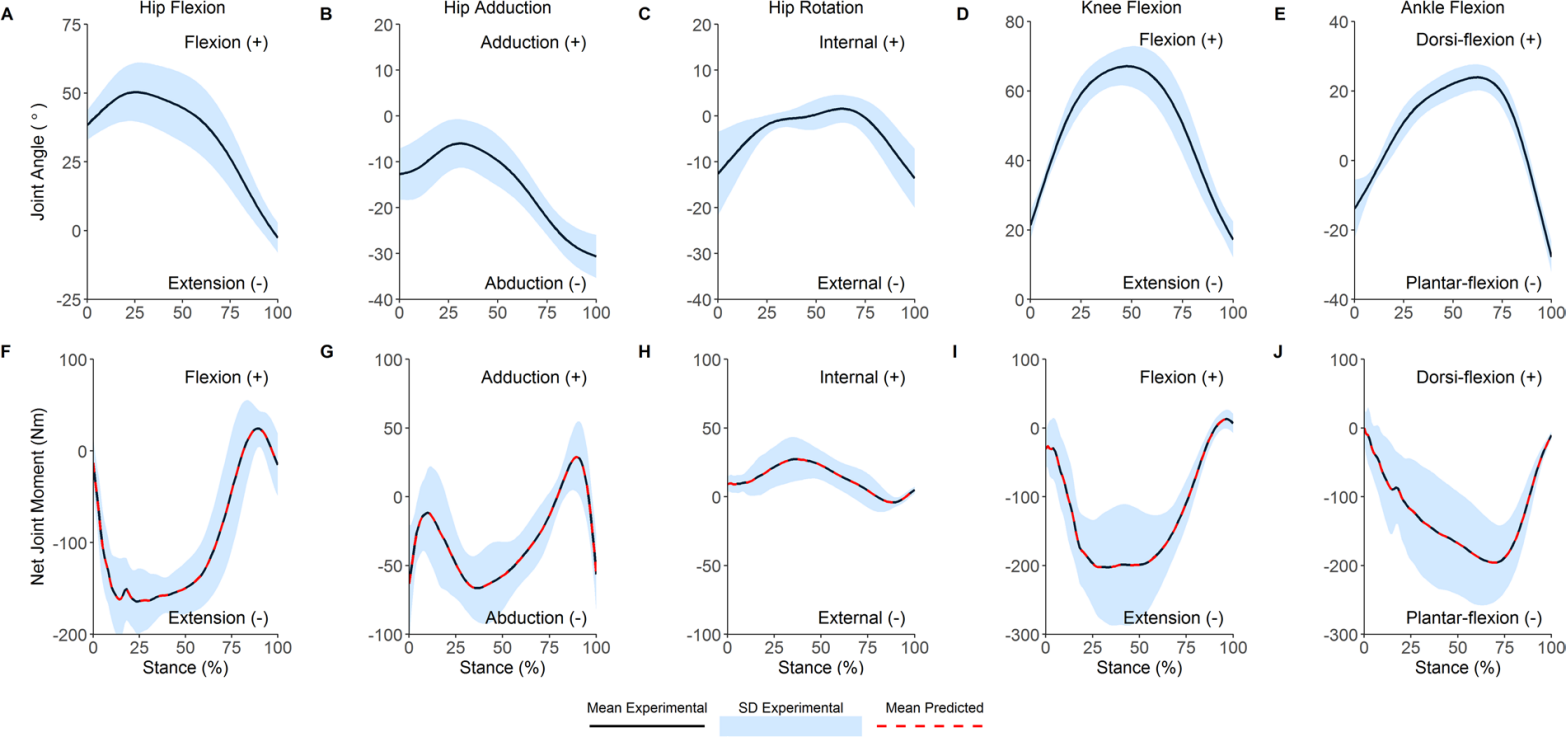
Joint angles and joint moments during the stance phase of the 45° unanticipated sidestep cut. Top row, mean (black line) and SD (blue shaded) joint angles; bottom row, experimental (mean, black line; SD, shaded blue) and predicted (red dotted) lower-limb joint moments.

To provide confidence in our simulations, we performed qualitative comparisons between the model-based predicted activations and experimental EMG data, accounting for appropriate physiological delays (~100ms) as per current recommendations^34^. We obtained EMG data from experimental recordings conducted in the present study and from available data in the literature^35,36^. Since these comparisons were conducted to assess how well the simulation replicated the coordination pattern observed experimentally, the normalised EMG data were averaged across participants and then renormalised to the peak amplitude of each muscle. The predicted activations were processed using the same normalisation procedure as the EMG data, prior to these comparisons. We also compared the time-varying characteristics of our experimental joint angles and inverse dynamics based joint moments (Fig. 1) to ensure they were within 2 SD of prior published data^34^. These qualitative comparisons were conducted across the entire stance phase because the weight acceptance phase was generally too short to allow any firm conclusions to be made about how well our model-based predicted data temporally matched experimental data as well as data obtained from the literature.

We quantitatively verified that our muscle-derived joint moments (computed from the predicted muscle forces and their respective moment arms) matched the experimentally measured joint moments (computed via inverse dynamics) by calculating the normalised root-mean-square error (nRMSE) and coefficient of determination (R^2^). The nRMSE was calculated as:

1 $$\mathrm{nRMSE}\,( \% )=100\,\times \,\frac{\sqrt{\frac{{\sum }_{i=1}^{n}{(Experimental{}_{i}-Predicte{d}_{i})}^{2}}{n}}}{\max (Experimental)-\,{\rm{\min }}(Experimental)}$$

To verify the suitability of the foot-ground contact model, superposition errors between experimental and simulated ground reaction forces were quantitatively evaluated via computation of the nRMSE and R^2^. These data were reported as the median and interquartile range (IQR) due to non-normal distributions.

### Data Availability

The datasets generated and/or analysed during the current study are available from the corresponding author on reasonable request.

## Results

### Validation

**Figure 2:**
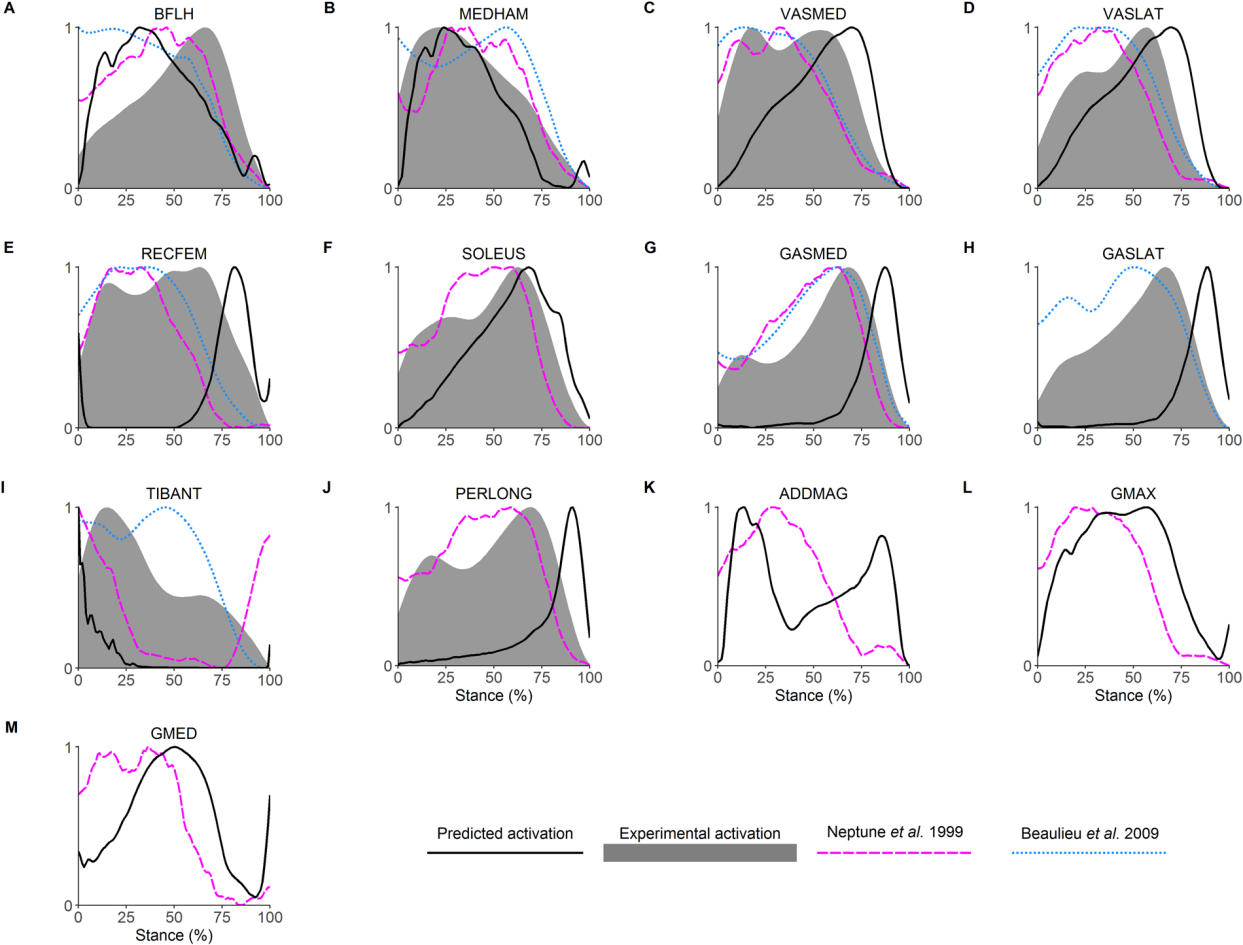
Comparison of predicted (black line) and experimental activations (grey shaded) from the current data during the stance phase of the 45° unanticipated sidestep cut. Literature reference activations, magenta dashed line, Neptune *et al*.^35^; blue dotted line, Beaulieu *et al*.^36^. BFLH, biceps femoris long head; MEDHAM, medial hamstrings (semitendinosus and semimembranosus); VASMED, vastus medialis; VASLAT, vastus lateralis; RECFEM, rectus femoris; SOLEUS, soleus; GASMED, gastrocnemius medialis; GASLAT, gastrocnemius lateralis; TIBANT, tibialis anterior; PERLONG, peroneus longus; ADDMAG, adductor magnus; GMAX, gluteus maximus; GMED, gluteus medius.

Muscle-derived joint moments showed excellent agreement with inverse dynamics based joint moments (R^2^, 1.0, IQR, 1.0 to 1.0; nRMSE, 3.2 × 10^−3^%, IQR, 1.5 × 10^−3^ to 1.1 × 10^−2^%; Fig. 1). The foot-ground contact model also showed acceptable results, with model-predicted ground reaction forces in agreement with experimentally measured ground reaction forces (R^2^, 0.95, IQR, 0.92 to 0.97; nRMSE, 7.9%, IQR, 6.1 to 10%). Additionally, once appropriate physiological delays were taken into account (100 ms corresponds to ~25% of stance phase), reasonable agreement was evident between the predicted muscle activations from the model and experimentally recorded EMG data obtained from the current study as well as prior literature (Fig. 2).

### Anteroposterior shear joint reaction force

**Figure 3:**
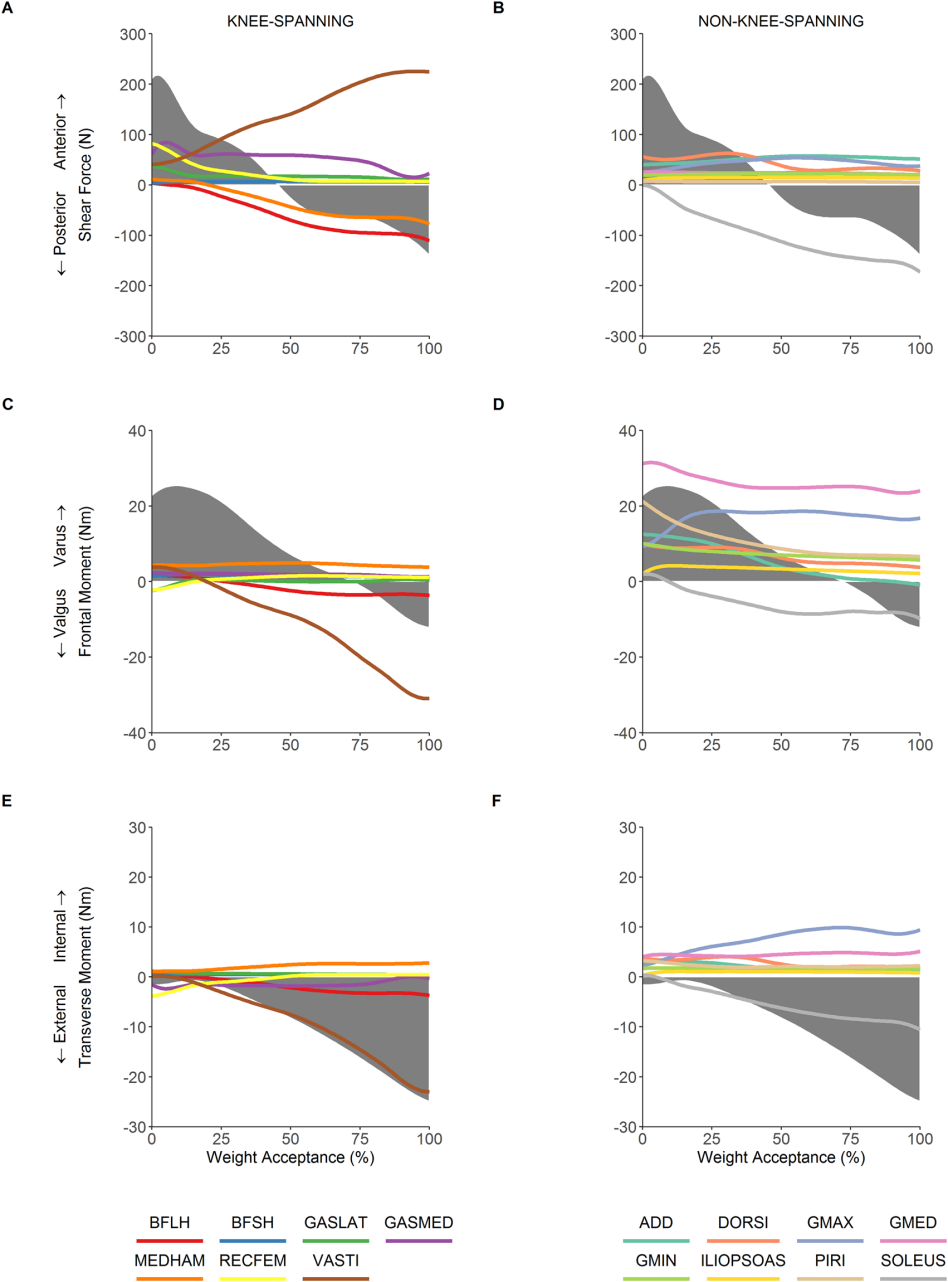
Muscular contributions to knee anteroposterior shear joint reaction force (row 1), frontal plane knee joint reaction varus/valgus moment (row 2) and transverse plane knee joint reaction internal/external rotation moment (row 3) during the weight acceptance phase of the 45° unanticipated sidestep cut. The first column (panels A, C and E) show knee-spanning muscles, the second column (panels B, D, and F) show non-knee-spanning muscles. Note that the shaded grey represents the experimental value (net value accounting for all forces) for each reaction load. BFLH, biceps femoris long head; BFSH, biceps femoris short head; GASLAT, gastrocnemius lateralis; GASMED, gastrocnemius medialis; MEDHAM, medial hamstrings (semitendinosus and semimembranosus); RECFEM, rectus femoris; VASTI, vasti; ADD, adductors (adductor brevis, longus and magnus); DORSI, dorsiflexors (tibialis anterior, extensor digitorum and hallucis longus), GMAX, gluteus maximus; GMED, gluteus medius; GMIN, gluteus minimus; ILIOPSOAS, iliopsoas (iliacus and psoas major); PIRI, piriformis; SOLEUS, soleus.

The net anteroposterior shear force was characterised by an anterior shear force of 218 N at initial contact, which gradually declined until switching to a posterior shear force at 46% of the weight acceptance phase (Fig. 3A and B). The greatest contributors to the posterior shear force were the biarticular hamstrings and soleus. The contribution of each of these muscles increased throughout weight acceptance, peaking at 173N, 111N, and 77N for the soleus, biceps femoris long head and medial hamstrings, respectively. The anterior shear force was primarily produced by the quadriceps and gastrocnemius muscle groups. The vasti’s contribution increased throughout weight acceptance, peaking at 225N, whilst contributions from the rectus femoris and lateral gastrocnemius peaked at initial contact at 83N and 38N, respectively. The medial gastrocnemius peaked at 84N at 5% of weight acceptance, and remained at around 60N for the majority of the remainder weight acceptance. The non-knee-spanning ankle dorsi-flexors (tibialis anterior, extensor digitorum and hallucis longus), adductors and gluteus maximus also contributed 50–60N during mid-weight acceptance. The shift to a net posterior shear force at 46% of weight acceptance was mainly explained by a decline in the contribution from the gastrocnemius towards anterior shear, and an increase in the contribution from the biarticular hamstrings and soleus towards posterior shear.

### Frontal plane joint reaction moment (varus/valgus)

A varus knee joint reaction moment (peak of 25 Nm) was present for the first 72% of weight acceptance, whereas a valgus knee joint reaction moment (peak of 12 Nm) was present for the remaining portion (Fig. 3C and D). Throughout weight acceptance, the gluteal muscles had the greatest capacity to oppose the valgus moment. The gluteus medius produced the largest varus moment (ranging from 23–32 Nm across weight acceptance). Substantial contributions were also made by the piriformis (7–21 Nm) and gluteus maximus (9–19 Nm). The transition to a valgus knee joint reaction moment was driven by decreasing contributions from the gluteals, piriformis and adductors towards a varus moment, and increasing contributions from the vasti (up to 31 Nm), soleus (up to 10 Nm) and biceps femoris long head (up to 4 Nm) towards a valgus moment.

### Transverse plane joint reaction moment (internal/external rotation)

An external rotation knee joint reaction moment was present throughout the entire weight acceptance period (Fig. 3E and F). The external rotation moment was 1–2 Nm for the first quarter of weight acceptance. It progressively increased during the second half of weight acceptance, peaking at 25 Nm. The dominant contributors towards this moment were the vasti (up to 23 Nm) and soleus (up to 10 Nm) muscles. The gluteus maximus (2–10 Nm) and gluteus medius (4–5 Nm) muscles had the greatest potential to oppose this moment (i.e. contribute to an internal rotation knee joint reaction moment) throughout weight acceptance.

## Discussion

This study has shown that both knee-spanning and non-knee-spanning muscles contribute to the tibiofemoral reaction forces and moments during the weight acceptance phase of a rapid unanticipated sidestep cut. Notably, we found the biarticular hamstrings and the soleus muscles to have the greatest potential to oppose the anterior shear reaction force, whilst the hip abductors (gluteus medius, gluteus maximus and piriformis) had the greatest potential to oppose the knee valgus reaction moment. To the authors’ knowledge, no previous studies have calculated muscular contributions to knee joint loads during a rapid unanticipated sidestep cut.

The data reported in the present paper are largely consistent with prior literature. Experimental kinematics (Fig. 1, top row) and inverse dynamics based joint moments (Fig. 1, bottom row) were within 2 SD of prior research investigating similar cutting tasks^18,37,38^. Additionally, the predicted muscle activations showed reasonable agreement with EMG data for sidestep cutting obtained from the current study and the literature^35,36^. Whilst this consistency provides some evidence that our simulations were physiologically acceptable, the main focus of the present study concerned muscular contributions to the tibiofemoral anteroposterior shear reaction force as well as the frontal and transverse plane joint reaction moments. To our knowledge, only one study by Sritharan and colleagues^33^ has reported comparable data. They computed the muscular contributions to the ‘external’ knee varus moment during gait. Note that Sritharan *et al*.^33^ quantified the muscular contributions to the inverse dynamics based joint moments, rather than the joint reaction forces/moments as we have reported here. Additionally, they did not include all of the muscles we have evaluated in the present study. Finally, they investigated walking, which has quite different biomechanical demands to sidestep cutting. Nevertheless, some consistent functional roles for key muscles are evident when comparing data from Sritharan *et al*.^33^ with equivalent data from the present study. For example, we observed that the gluteal muscle group had the greatest potential to generate a varus knee joint reaction moment during the weight acceptance phase of sidestep cutting, i.e. these muscles opposed the net valgus knee joint reaction moment that occurred during the final 25% of weight acceptance (Fig. 3D). Similarly, Sritharan *et al*.^33^ found that the gluteus medius and maximus muscles were the major contributors to the ‘external’ knee varus moment during the stance phase of walking. They also found that the vasti and soleus were dominant contributors towards an ‘external’ knee valgus moment, which is consistent with the findings from the current study for cutting (Fig. 3C and D).

### Anteroposterior shear joint reaction force

The primary role of the ACL is to resist anterior tibial translation^4^, and thus tibiofemoral shear has received much attention in the literature^5–7^. The quadriceps and hamstring muscle groups are of particular interest in this respect due to their ability to induce anterior and posterior shear forces, respectively^39^. We found that the vasti and biarticular hamstrings were indeed major contributors to anterior and posterior shear forces, respectively. However, our analysis provided insight into the critical role of other muscles, particularly the gastrocnemius and soleus, which appeared to have considerable yet opposing roles in the development of tibiofemoral shear force (Fig. 3A and B). The opposing roles of the gastrocnemius and soleus muscles has been observed in previous research investigating contributions to trunk and leg segmental energy^40^ as well as whole body sagittal plane angular momentum during gait^41^. We have shown that the soleus tends to induce posterior shear reaction forces, whilst the gastrocnemius tends to induce anterior shear reaction forces at the tibiofemoral joint. Our results therefore suggest that the soleus and gastrocnemius represent ACL agonists and antagonists, respectively; an observation that is consistent with prior musculoskeletal modelling^27,42^ and *in-vivo* studies^43^. In contrast to these findings, Morgan and colleagues^31^ reported that the gastrocnemius plays a role in unloading the ACL by increasing joint compression and thereby resisting tibial translation^44^. However, this assertion was based on a hypothetical explanation of elevated gastrocnemius forces observed in participants with low versus high estimated ACL loading. The role of joint compression is contentious, as animal models have shown that whilst joint compression may act to reduce anteroposterior translation, the direct influence on ACL loading may still be hazardous^45^. Nevertheless, we accept that muscular contributions to knee joint compression could have potential implications for ACL loading, thus we have computed these values for completeness and have included the results as supplementary material for the interested reader (see Supplementary Fig. S2). In contrast, knee joint anterior shear force has consistently been associated with ACL loading^5,7,46–49^, thus our cause-effect analysis showing that the gastrocnemius group induces anterior shear forces would suggest that the role of the gastrocnemius on its own is unlikely to be “protective”.

### Frontal and transverse plane joint reaction moments

One of the most noteworthy findings in this study is that the gluteal muscle group is capable of generating a varus knee joint reaction moment, thus opposing (or controlling the magnitude of) the net valgus knee joint reaction moment during the final 25% of weight acceptance of sidestep cutting. The gluteus medius provided the greatest contribution to the varus knee joint reaction moment for the entire weight acceptance phase, whilst other muscles (piriformis and gluteus maximus and minimus) also made appreciable contributions (Fig. 3D). This result has implications for preventative and rehabilitative interventions, as both knee valgus loading^50^ and lower hip abduction strength^51^ have been prospectively associated with ACL injury. Additionally, knee valgus loading has been observed during ACL injuries^9–11^, and has been directly related to ACL loading^5,7,8^. The gluteal muscles were also found to be the primary contributors to the internal rotation knee joint reaction moment (Fig. 3F), which potentially increases loads on the ACL^5,7,12^. However, the size of this contribution was relatively small (<10 Nm), and the tibiofemoral joint never experienced a net internal rotation reaction moment at any stage during the weight acceptance phase (Fig. 3E and F). As sidestep cutting is typically associated with valgus loading^32^, which is thought to be particularly relevant for non-contact injury mechanisms^9,20^, the function of the gluteal muscle group may be an important target for prevention programs aiming to reduce ACL injury risk. To our knowledge, no other study has demonstrated the importance of the gluteus medius (or the other hip abductor muscles) for opposing the knee valgus moment that occurs during sidestep cutting.

### Simultaneous multi-direction loading

It is thought that loads on the ACL are greatest when the knee joint is exposed to an anterior shear force together with a valgus and an internal rotation moment^5,7,46^. Whilst this specific combination of tibiofemoral reaction forces and moments was not observed to occur simultaneously in our data (Fig. 3), muscular contributions must still be considered across multiple planes due to their potential to cause or oppose relevant joint reaction forces and moments. Whilst a valgus moment that occurs together with an internal rotation moment has the potential to increase load on the ACL^5,7,8,12^, none of the major contributors to a valgus knee joint reaction moment were also found to be major contributors to an internal rotation knee joint reaction moment (Fig. 3C–F). The relative importance of non-sagittal loads to ACL loading is not universally accepted^52^, whereas anterior and posterior shear force have been consistently shown to load and unload the ACL, respectively^5–7,46–49^. Subsequently, appropriate muscular targets for interventions should be chosen primarily based on the magnitude of their contributions to anteroposterior shear force, with contributions to non-sagittal plane joint reaction moments perhaps a secondary consideration.

### Key clinical implications

Based on the findings from this study, we suggest that injury prevention strategies should focus on optimising the function of the hamstring muscle group, as the biceps femoris long head and medial hamstrings were shown to be the two primary contributors to posterior shear during weight acceptance of sidestep cutting (Fig. 3A). Additionally, these muscles induce opposite loading patterns in the frontal (Fig. 3C) and transverse planes (Fig. 3E), thus reducing the likelihood for combined unfavourable loading patterns to be generated. The function of the soleus would also seem important, due to this muscle’s contribution to the posterior shear knee joint reaction force (Fig. 3B), whilst also contributing to an external rotation knee joint reaction moment (Fig. 3F). However, from a practical standpoint, the function of the soleus may be difficult to isolate from the gastrocnemius, a muscle group which we found to contribute to an anterior shear reaction force at the knee. Finally, the gluteal group, especially the gluteus medius and the piriformis muscles, were the dominant controllers of the valgus knee joint reaction moment (Fig. 3D), and also made no meaningful contribution towards anterior shear and their contribution towards an internal rotation knee joint reaction moment was minimal. For these reasons, we consider training the function of the gluteus medius and piriformis muscles to be of high priority in ACL prevention programs.

### Limitations

Whilst our study has revealed some novel insights, we acknowledge that there are some limitations to this work. One limitation is that the present study only involved a cohort of eight healthy recreationally active males. Further research should consider the influence of different populations such as females, specific athletic subgroups, and pathological populations. Additionally, participants were barefoot during the performance of the sidestep cut, which is not representative of many sports that involve footwear. There is the possibility that this may have resulted in an imposed foot-strike pattern for some participants, and a natural foot-strike pattern for others^53^. However, we do not believe this influenced the conclusions of the study. The advantage of the barefoot condition was that it ensured a consistent foot-ground interaction across participants, and allowed exposure of the foot for marker placement.

Another limitation is that we did not compute ACL forces directly. Whilst including knee ligaments into the musculoskeletal model would have allowed us to predict ligament (or ACL) forces directly, this complexity would come at the cost of introducing additional uncertainties related to *in-vivo* ligament properties^54^. Due to the sensitivity of estimated ACL forces to these ligament properties (e.g. reference strains and ligament stiffness)^54^, we opted to exclude ligaments from the model.

The decision to exclude ligaments from the model meant that translations and non-sagittal rotations at the knee needed to be constrained as a function of the knee flexion angle^23^, similar to prior studies^27^, in order to ensure our predicted muscle forces were as accurate as possible. Another advantage of adopting such constraints is minimising the impact of soft tissue artefact. Prior research has shown that non-sagittal plane knee rotations are particularly sensitive to soft tissue artefact when using skin-mounted marker systems^18^. Whilst soft tissue artefact can influence all joint angles, we used a global optimisation inverse kinematics algorithm to obtain our joint angle data, which has previously been shown to be capable of minimising the influence of soft tissue artefact^24^. We note that our kinematic data are consistent with prior literature investigating similar change of direction tasks using both skin-mounted^37,38^ and bone-pin marker systems^18^.

Muscle forces in the present study were estimated using a static optimisation algorithm, which does have some limitations. Unfortunately, muscle forces cannot be directly validated, as *in-vivo* muscle forces are not practically feasible to measure^55^, thus we have no way of directly validating our model predictions. Static optimisation has been shown to provide accurate predictions of *in-vivo* joint contact forces^56,57^, which serves as an indirect validation of muscle forces due to the high dependency of joint contact forces on muscle forces^55^. Furthermore, our predicted muscle activations showed reasonable agreement with experimentally recorded EMG data across the stance phase (Fig. 2). It has been suggested that static optimisation may not adequately predict co-contraction of muscles. However, our predicted muscle activations (Fig. 2) as well as recently published data^26^ do display evidence of co-contraction. Nevertheless, we recognise that these co-contraction patterns were not necessarily subject-specific but we do not believe this limitation influenced our conclusions. Further research utilising more subject-specific approaches, such as EMG-driven and EMG-hybrid modelling^58,59^, may yield further clinical insight.

## Conclusion

In conclusion, this study demonstrated that knee-spanning as well as non-knee-spanning muscles contribute substantially to anteroposterior shear forces as well as frontal and transverse plane joint reaction moments at the tibiofemoral joint. Specifically, the vasti and gastrocnemius muscles were found to be the major contributors to the anterior shear reaction force, whilst the biarticular hamstrings and the soleus were the major contributors to the posterior shear reaction force. The valgus knee joint reaction moment was primarily produced by both knee-spanning (vasti and biceps femoris long head) and non-knee-spanning (soleus) muscles. This moment was opposed by the non-knee-spanning gluteal muscles, particularly the gluteus medius, gluteus maximus and piriformis. The external rotation knee joint reaction moment throughout the weight acceptance phase of sidestep cutting was primarily generated by the vasti and soleus muscles. Based on our consideration of multiple loading states, we conclude that the hamstrings (biceps femoris long head and medial hamstrings), soleus, and the gluteals (especially gluteus medius) have the greatest potential to offset ACL loading during an unanticipated sidestep cutting task. Optimising the function of these muscles should therefore be of high priority in rehabilitative and preventative programs.

## Electronic supplementary material

Supplementary Information
