# Supplementary material for: Non-knee-spanning muscles contribute to tibiofemoral shear as well as valgus and rotational joint reaction moments during unanticipated sidestep cutting
Source: Sci Rep. 2018 Feb 6;8:2501. doi: 10.1038/s41598-017-19098-9 (PMC5802728; doi:10.1038/s41598-017-19098-9)
Supplement: Supplementary file 1 — Supplementary Information [file 41598_2017_19098_MOESM1_ESM.pdf]

Non knee-spanning muscles contribute to tibiofemoral shear as well as valgus and rotational joint reaction moments during unanticipated sidestep cutting

Authors

Nirav Maniar[1], Anthony G Schache[2], Prasanna Sritharan[2,3], David A Opar[1]

[1]School of Exercise Sciences, Australian Catholic University, Melbourne, Australia

[2]Department of Mechanical Engineering, The University of Melbourne, Melbourne, Australia

[3]Sports and Exercise Medicine Research Centre, La Trobe University, Melbourne, Australia

Supplementary Table S1. Functional groups of musculotendinous actuators used in the present study.

Functional group	Muscles	Musculotendinous actuators*
ADD	Adductor brevis	addbrev
	Adductor longus	addlong
	Adductor magnus	addmagProx
		addmagMid
		addmagDist
		addmagIsch
DORSI	Extensor digitorum longus	edl
	Extensor hallucis longus	ehl
	Tibialis anterior	tibant
GMAX	Gluteus maximus	glmax1
		glmax2
		glmax3
GMED	Gluteus medius	glmed1
		glmed2
		glmed3
GMIN	Gluteus minimus	glmin1
		glmin2
		glmin3
ILIOPSOAS	Iliacus	iliacus
	Psoas major	psoas
MEDHAM	Semimembranosus	semimem
	Semitendinosus	semiten
VASTI	Vastus Intermedius	vasint
	Vastus Lateralis	vaslat
	Vastus Medialis	vasmed

*Actuator names taken from musculoskeletal model[22].

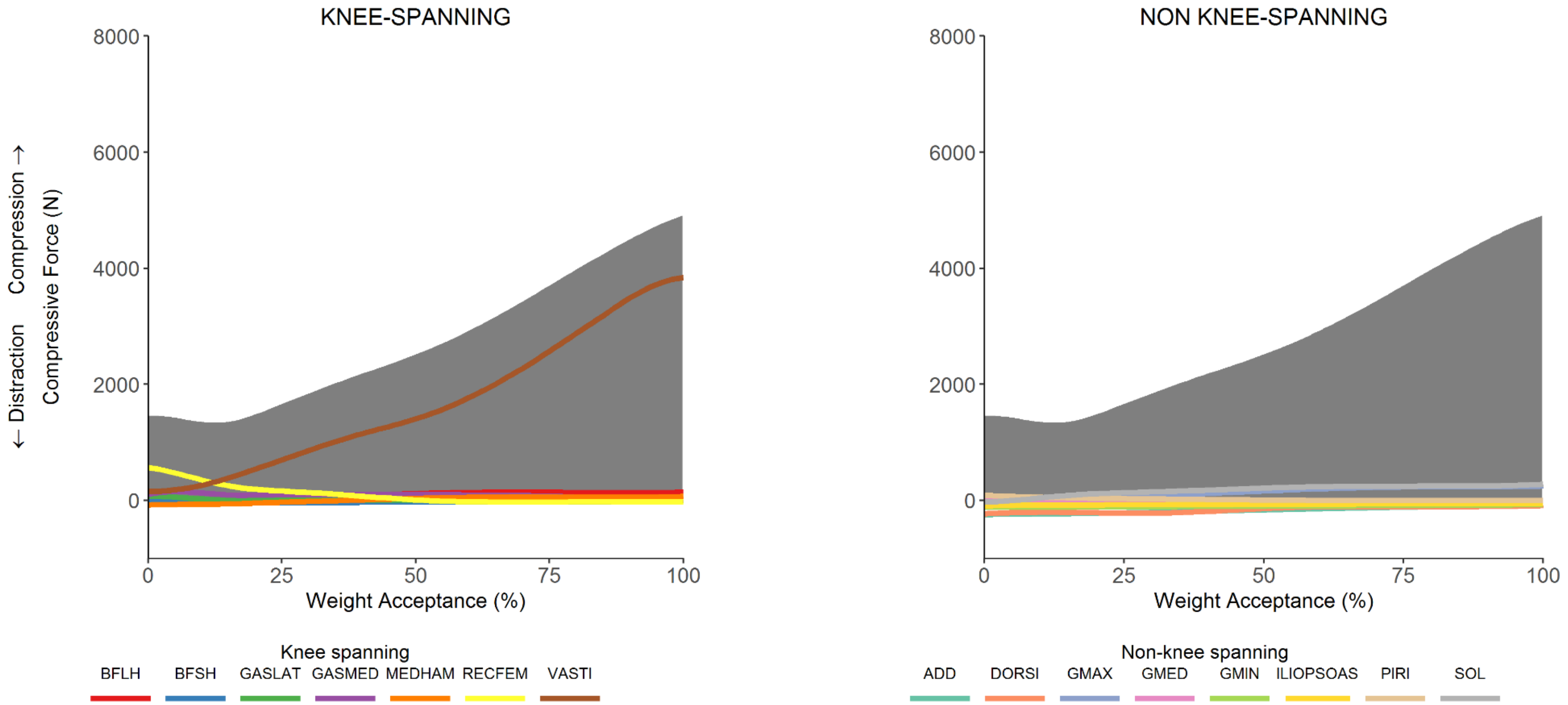

Supplementary Figure S2. Muscular contributions to knee compressive force during the weight acceptance phase of the 45º unanticipated sidestep cut. The first column shows knee-spanning muscles, the second column shows non knee-spanning muscles. Note that the shaded grey represents the experimental value (net value accounting for all forces) for each reaction load. BFLH, biceps femoris long head; BFSH, biceps femoris short head; GASLAT, gastrocnemius lateralis; GASMED, gastrocnemius medialis; MEDHAM, medial hamstrings (semitendinosus and semimembranosus); RECFEM, rectus femoris; VASTI, vasti; ADD, adductors (adductor brevis, longus and magnus); DORSI, dorsiflexors (tibialis anterior, extensor digitorum and hallucis longus), GMAX, gluteus maximus; GMED, gluteus medius; GMIN, gluteus medius; ILIOPSOAS, iliopsoas (iliacus and psoas major); PIRI, piriformis; SOL, soleus.
